# Effects of Gas Adsorption on the Mechanical Properties of Amorphous Polymer

**DOI:** 10.3390/polym11050817

**Published:** 2019-05-07

**Authors:** Shin Won Kim, Joo Seong Sohn, Hyun Keun Kim, Youngjae Ryu, Sung Woon Cha

**Affiliations:** School of Mechanical Engineering, Yonsei University, 50, Yonsei-ro, Seodaemun-gu, Seoul 03722, Korea; 0shinmy0@yonsei.ac.kr (S.W.K.); ssamjjang87@yonsei.ac.kr (J.S.S.); sagegny@yonsei.ac.kr (H.K.K.); yjryu1027@yonsei.ac.kr (Y.R.)

**Keywords:** polymer, amorphous region, carbon dioxide, polymer-gas mixture, impact strength, tensile strength, gas adsorption, network density

## Abstract

This study investigates the properties of a polymer–gas mixture formed through diffusion, based on the changes in the partial pressure and observed changes in the impact and tensile strengths owing to the gas dissolution. The high-pressure gas dissolves into a solid-state polymer through diffusion based on the difference in the partial pressure. This dissolved gas is present in the amorphous region within the polymeric material temporarily, which results in the polymer exhibiting different mechanical properties, while the gas remains dissolved in the polymer. In this study, the mechanical properties of amorphous polyethylene terephthalate (APET) specimens prepared by dissolving CO_2_ using a high-pressure vessel were investigated, and the resulting impact and tensile strengths were measured. These experiments showed that the increase in sorption rate of CO_2_ caused an increase in the impact strength. At 2.9% CO_2_ absorption, the impact strength of APET increased 956% compared to that of the reference specimen. Furthermore, the tensile strength decreased by up to 71.7% at 5.48% CO_2_ sorption; the stress–strain curves varied with the gas sorption rate. This phenomenon can be associated with the change in the volume caused by CO_2_ dissolution. When the APET absorbed more than 2.0% CO_2_ gas, sample volume increased. A decrease in the network density can occur when the volume is increased while maintaining constant mass. The CO_2_ gas in the polymer acted as a cushion in impact tests which have sorption rates above 2%. In addition to the reduction in the network density in the polymer chain, Van Der Waals forces are decreased causing a decrease in tensile strength only while CO_2_ is present in the APET. These observations only occur prior to CO_2_ desorption from the polymer.

## 1. Introduction

In recent years, the use of polymers has been rapidly increasing due to their superior moldability, low cost, and beneficial physical properties. In particular, polymeric materials are extensively used in the production of general household goods and machinery parts, and have found applications in the medical fields. Processing techniques for producing such polymer products have progressed over the years; foaming technology has been developed as a manufacturing method for producing lightweight products. One example, the microcellular foaming process, initially developed in the 1980s at the Massachusetts Institute of Technology, USA, is a polymer processing technology that can lead to a material reduction, weight reduction, change in heat characteristic, change in sound absorption, and a sound insulation capability [1,2]. There is a difference between this process and conventional foaming using a chemical blowing agent in that an inert gas (CO_2_, N_2_, or Ar) in a supercritical fluid state is used as the blowing agent. The influx of such gases changes the behavior of the liquid-state polymer. Yoon and Cha confirmed experimentally that the dissolution of gas causes changes in the glass transition temperature and viscosity [3]. Pasricha and Kumar studied the changes in a polymer material by observing the creep behavior of polycarbonate (PC) owing to the gas dissolution [4].

**Figure 1:**
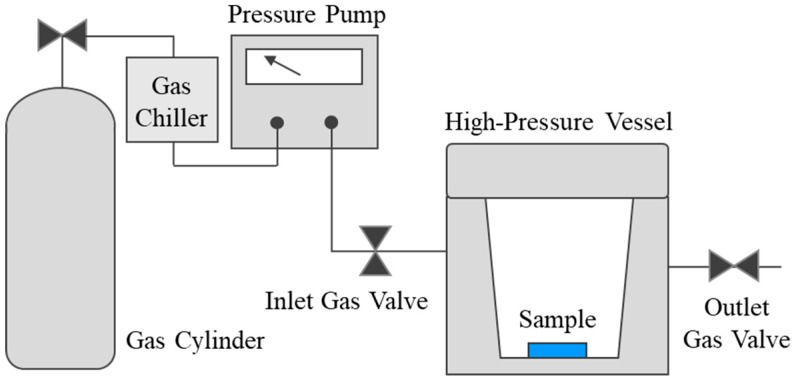
High-pressure vessel for batch foaming process.

As the demand for such polymers continues to increase, studies on their processing and material properties are ongoing. A gas under high pressure is dissolved into the solid-state polymeric material through diffusion based on the difference in partial pressure. By using this phenomenon, nanoparticles or powder-type pigments can be impregnated together with gas into a polymeric material. These can be used in many fields of industry, such as polymer dying, and in the production of new smart material by impregnating additives [5]. However, usage is still limited because of insufficient research concerning polymer-gas mixtures. Figure 1 illustrates the high-pressure vessel for the batch foaming process. There is a chamber for diffusing gas into the polymer sample. To maintain high pressure above bombe pressure, a pressure pump is connected between the bombe and chamber.

In this study, a solid-state polymer–gas mixture is formed through a batch foaming process, and the impact and tensile strengths, typical mechanical properties of polymeric materials, are considered. For this process, gases such as CO_2_, N_2_, Ar, and He are used. When a high-pressure condition no longer exists, a dissolved gas diffuses and disappears owing to the new equilibrium. That is, the polymer–gas mixture formed by exposing the polymeric material to high-pressure conditions is temporary and exhibits characteristics different from those of a conventional polymeric material in the presence of the gas. According to previous research, polymer–gas mixtures show a decrease in viscosity and a lower glass transition temperature when compared with those of conventional polymeric materials [6]. We investigated the mechanisms through which gas affects the polymer–gas mixture by identifying the influence of the gases present in the polymeric material by measuring the mechanical properties. As previously mentioned, the sorption of the gas takes place through diffusion based on the difference in partial pressure. Assuming that the dissolved gas is located in the non-crystalline regions of the polymeric material, and that the polymeric material does not undergo chemical changes during the dissolution of the gas, the sorption of the gas as determined through the following model can be deduced (Equation (1)).

(1) $$n=\frac{Nv}{V}=\left(\frac{P}{zRT}\right)\left[\alpha \cdot v\cdot f_{v}\left(1-Cr\right)\right]$$

Here, N is the number of gas molecules in a particular volume (V), and n is the number of gas molecules present in that volume (v) of the polymer sample. That is, the number of dissolved gas molecules is estimated based on the proportional formula, N:V = n:v, where P is the pressure of the dissolved gas, R is the gas constant, and T is the temperature. Because the pressure at which the gas dissolves is high, the compressibility (z) of the gas is considered in the ideal gas equation. In addition, because the gas flows into the free volume portion of the non-crystalline region, the term corresponding to the square bracket is included to estimate the free volume. This is represented by the product of the ratio of the amorphous region (1−Cr) to the free volume ($f_{v}$) and by the material constant (α) in the amorphous region of the total polymeric material. That is, the sorption rate of the gas varies depending on the type of the polymeric material, the degree of crystallization, and the free volume. On the extrinsic side of the material, it is affected by the temperature and pressure of the dissolved gas, which in turn changes the gas concentration [7,8,9,10,11].

The state of the polymer differs from a previously reported study. The plasticizing effect of CO_2_ is a well-known phenomenon [12]. However, the previous study concentrated on the liquid state because their research initialized from extrusion and injection molding [12,13]. In this study, a solid-state polymer is the target for developing a new polymer forming process. There are several methods to create a polymer from the solid-state and in those cases, a mechanical property, such as tensile strength, is an important consideration. This study explores the use of gas sorption in the development of a polymer forming process.

## 2. Materials and Methods

### 2.1. Material

**Figure 2:**
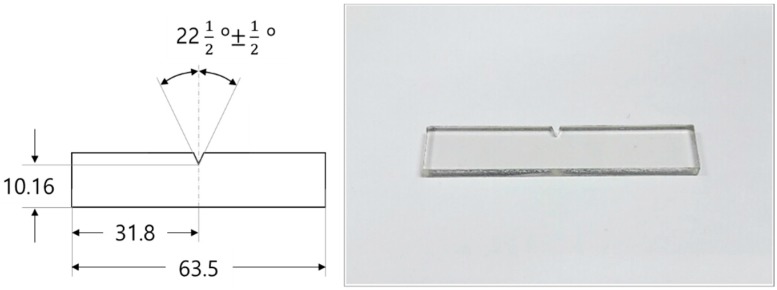
Dimensions and actual appearance of impact-strength specimen.

**Figure 3:**
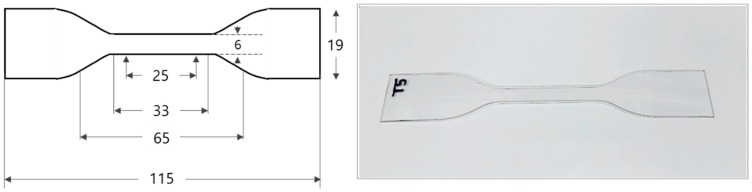
Dimensions and actual appearance of tensile-strength specimen.

The material used in this study was amorphous polyethylene terephthalate (APET, Taekwang Newtec, Inc. Seoul, Korea, Grade No. Plastar-100 sheet by extrusion). Impact strength specimens were prepared from an APET sheet with a thickness of 3 mm. A notch was used for measuring during the Izod impact test [14]. The shape and dimensions of the impact-strength specimen are shown in Figure 2. The tensile-strength specimens were sheets with a thickness of 0.8 mm, made in accordance with ASTM D638 Type-IV specifications [15]. When the prepared specimens were measured, the tensile strength of the APET was 63.48 MPa and the elastic modulus was 2.66 GPa. The shape and dimensions of the processed specimen are shown in Figure 3.

### 2.2. Method

#### 2.2.1. Experiment on Change in Impact Strength

**Figure 4:**
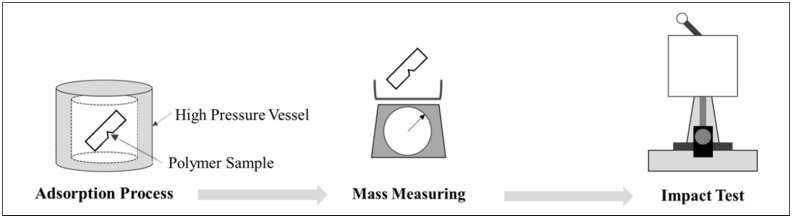
Experimental process for studying changes in impact strength through CO_2_ adsorption.

To investigate the change in impact strength due to the gas dissolution, we controlled the gas sorption rate by regulating the temperature and pressure during the batch foaming process and changing the exposure time (adsorption time) to a high-pressure condition (at least five samples per sorption condition). After high-pressure CO_2_ was dissolved in the APET specimen, the sorption rate was checked by measuring the mass of the specimen. Further, the change in volume owing to the dissolution of CO_2_ was determined by measuring the width, height, and length of the sample; the change pattern from the CO_2_ dissolution was then confirmed. The measurement of the impact strength (ASTM D256) [14] was conducted using a notched specimen with a digital impact tester (Salt Co., Ltd., Incheon, Korea, Model No. ST-120) (Figure 4).

#### 2.2.2. Experiment on Change in Tensile Strength

**Figure 5:**
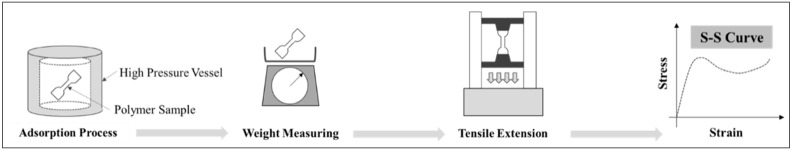
Experimental process for studying changes in the tensile strength through CO_2_ adsorption.

The pressure and temperature of the gas were regulated by a high-pressure vessel to control the amount of CO_2_ dissolved. After the saturating process was conducted to dissolve the gas under the high-pressure condition of the high-pressure vessel, the tensile properties of the polymer–gas mixture were observed, as shown in Figure 5. Once the saturation process had concluded, the weight was measured within 5 min to calculate the sorption rate of the gas. The sorption rate equation is as shown in Equation (2).

(2) $$\mathrm{Sorption}\;\mathrm{Rate}\left(\%\right)=\frac{weight\;of\;dissolved\;CO_{2}}{weight\;of\;polymer\;sample}\times 100=\frac{m_{s}-m_{0}}{m_{0}}\times 100$$

After establishing the sorption rate, the tensile properties were confirmed using a universal test machine (QMESYS Co., Ltd., Gunpo, Korea, Model No. QM-100T). The cross-head speed was 5 mm/min for the measurement of the tensile strength (ASTM D638) [15], and the maximum tensile strength, elastic modulus, and elongation percentage of the material were confirmed based on the measured values.

### 2.3. Experimental Conditions

#### 2.3.1. Experiment on Change in Impact Strength

In this experiment, factors affecting the dissolution of CO_2_ were the temperature, pressure, and time. The temperature can directly affect the mechanical properties of APET as an amorphous polymeric material. Therefore, the experiment was conducted at 23 °C. To control the sorption rate of the gas, the pressure was fixed at 5 MPa and the adsorption time was manipulated. The conditions of the experiment are shown in Table 1.

#### 2.3.2. Experiment on Change in Tensile Strength

In polymer–gas mixtures, the most important parameter is the sorption rate. Methods for controlling the sorption rate include manipulating the CO_2_ pressure, time of exposure of the polymer specimens to high-pressure conditions, temperature at exposure, and the desorption time. Trials were carried out by fixing the other conditions while varying the exposure time to the high-pressure carbon dioxide. After the pressure is released, CO_2_ in the polymer is under a desorption condition (even while measuring mechanical property). Table 2 shows the experimental conditions.

## 3. Results and Discussion

### 3.1. Changes in Impact Strength by CO_2_ Adsorption

**Figure 6:**
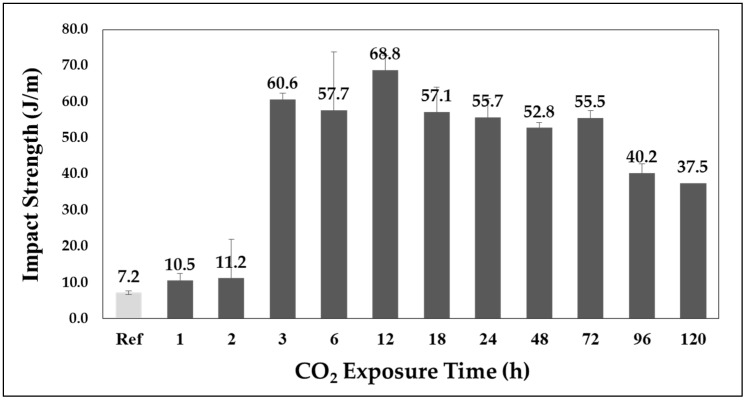
Impact strength of CO_2_ dissolved APET.

**Figure 7:**
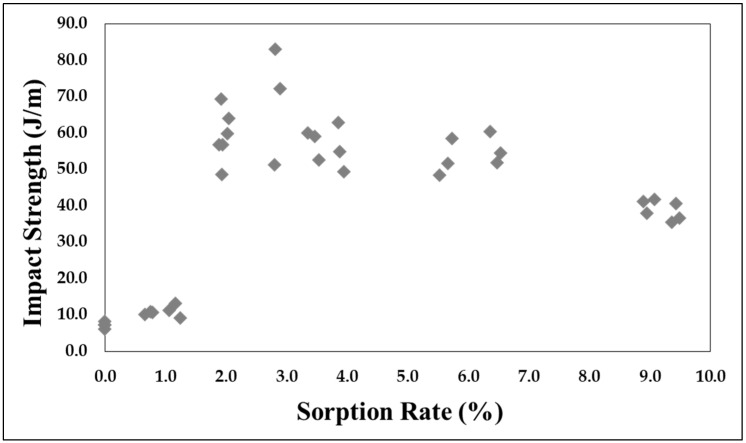
Relationship between impact strength and sorption rate.

Figure 6 and Figure 7 shows the changes in impact strength based on the CO_2_ adsorption time and sorption rate. While no significant trend is apparent, a critical point can be observed. The effect of the increase in impact strength is insufficient until an adsorption time of 2 h, at which a sorption rate of 1.13% occurs. However, the adsorption time of 3 h, which has a sorption rate of 2%, shows a rapid increase in strength. In addition, the maximum value of 68.8 J/m appears at an adsorption time of 12 h with a sorption rate of approximately 2.85%. This is an increase of approximately 956% as compared to that of the reference specimen. In addition, the sorption rate decreases again after the maximum value is reached. The impact strength profile according to the sorption rate is shown in Figure 7.

**Figure 8:**
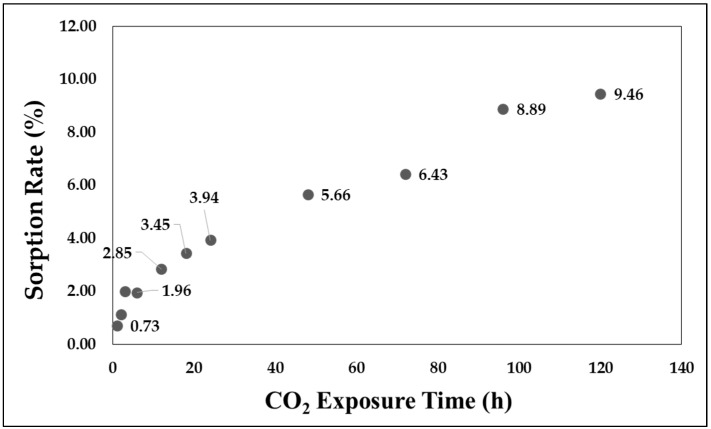
Adsorption rate according to adsorption time.

**Figure 9:**
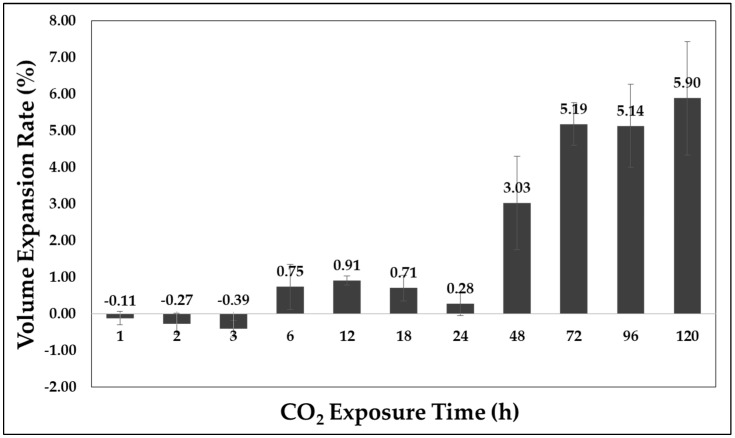
Volume expansion ratio according to adsorption time.

The sorption rate and volume expansion ratio of APET owing to CO_2_ dissolution are shown in Figure 8 and Figure 9, respectively. A change in sorption rate was monitored until 9.46% was achieved. It is clear that a dissolution occurs more rapidly during the early stage of adsorption.

**Figure 10:**
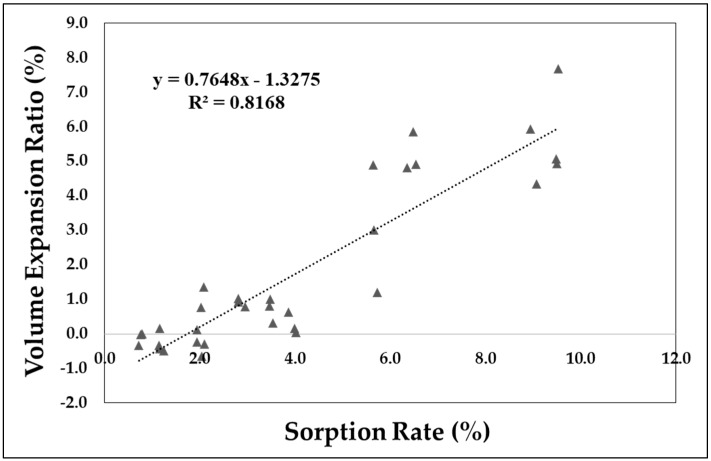
Relationship between adsorption rate and volume expansion ratio.

A change in volume is not observed in the section with an adsorption rate of 2% or less (adsorption time of 3 h), but a change does occur after that. Figure 10 shows a weak correlation between the adsorption rate and volume change. From this, a linear relationship is shown between the adsorption rate and volume expansion rate after the section with approximately 2% adsorption rate.

Based on the observed changes in the impact strength and volume expansion ratio, the point at which the impact strength increases and the point at which a change in volume starts to occur coincide with the region where the sorption rate is approximately 2%. That is, the abrupt change in intensity is closely related to the volumetric change owing to the CO_2_ dissolution [16]. First, when analyzed based on the results of volumetric changes according to sorption rate, there is no volume change in regions with less than 2% sorption rate. This is closely related to the fact that the conventional gas dissolution enters the free volume of the amorphous region of the polymeric material, and a 2% sorption rate can be an indirect indicator of the free volume. According to previous research, free volume only can be estimated, not measured directly [17]. Dissolving gas into a polymer sample provides a method to measure free volume. However, there are several factors, which influence volume expansion (including swelling of the polymer by sorption of CO_2_); more research is needed to support using this method for measuring free volume. Furthermore, it is deduced that the increase in impact strength is caused by the cushioning effect of the dissolved gas while expanding the volume [18]. Until 2% sorption rate, the polymer chains remained in their original position because no volume change occurred. However, after 2% sorption rate, the polymer chains are shifted by the dissolved gas which causes volume expansion. These results are similar to those observed with the plasticizing effect of CO_2_ on a polymer [19]. Due to this outcome and the tensile strength reduction by dissolved gas, the plasticizing effect of CO_2_ is still effective even in the solid-state polymer. Also, from a point above a certain sorption rate (2.85%), dissolved CO_2_ becomes a form of void (cell nucleation) that promotes crazing, and the strength begins to decrease again.

### 3.2. Changes in Tensile Strength by CO_2_ Adsorption

**Figure 11:**
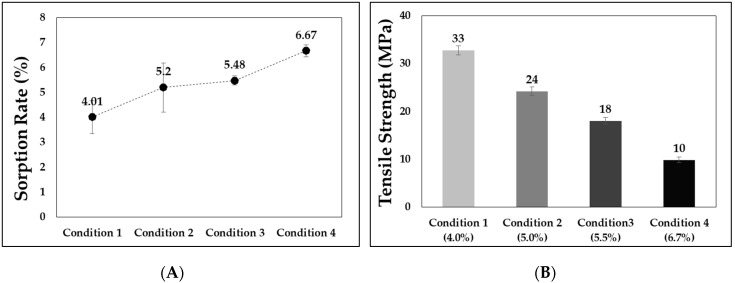
(**A**) Sorption rate and (**B**) tensile strength based on sorption rate.

Based on the experimental conditions used in this study, the sorption rate and tensile strength over time are: Conditions 1, 2, 3, and 4 correspond to 4, 8, 12, and 16 h of sorption time, respectively. The sorption rate increases with an increase in the sorption time (Figure 11A). At the same time, the larger the amount of gas dissolved, the greater the decrease in tensile strength that occurs. Figure 11B shows that Condition 1 (in which the gas is dissolved at 4.01%) reduces the tensile strength by 48.4%; Condition 2 (in which the gas is dissolved at 5.20%) decreases the tensile strength by 61.7%; and Condition 3 (in which the gas is dissolved at 5.48%) decreases the tensile strength by 71.7%.

**Figure 12:**
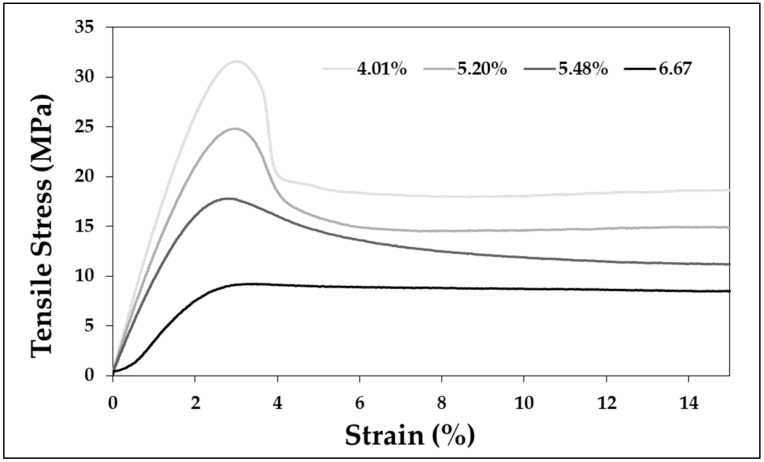
Stress–strain curve change of APET based on saturation time.

**Figure 13:**
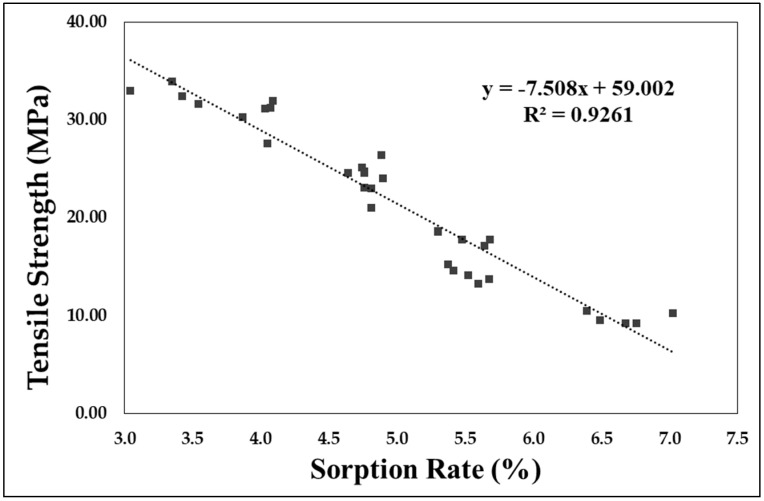
Tensile strength of APET based on CO_2_ sorption rate.

In addition to the tensile strength, the stress–strain curve for each condition is shown in Figure 12. As indicated in Figure 13, the strength corresponding to maximum tensile strength decreases with an increase in the gas sorption rate, as shown through the change in tensile strength. The largest difference is the change in strain softening in the stress–strain curve (strain softening is the term right after the peak, where the tensile stress is decreased). As the amount of gas dissolved increases, the slope of the strain softening section decreases after necking. Necking does not occur above the certain sorption rate (6.67%), and the strain owing to the tensile stress is converted from an inhomogeneous deformation to a homogenous deformation. In addition to the specified conditions, the change in tensile strength according to the sorption rate shows the pattern indicated in Figure 13. Figure 13 shows the tensile strength according to sorption rate for more than 30 specimens. From this figure, a change in tensile strength according to the sorption rate appears in a linear fashion, and the tensile strength decreases as the sorption rate of the gas increases. The formula related to this is shown in Equation (3).

Tensile strength (MPa) = −7.508 × (sorption rate(%)) + 59.002
(3)

**Figure 14:**
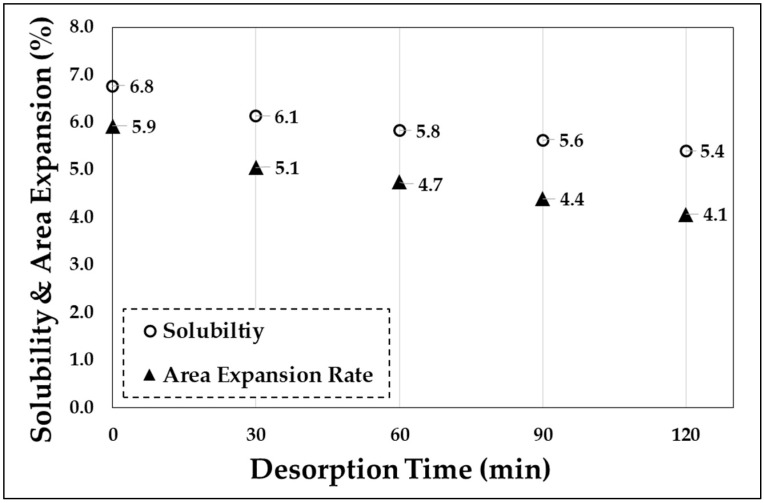
Sorption rate and area expansion based on the desorption time.

The cause of this property change can be deduced from the change in network density (density of the polymer chain network) for the gas dissolution. In fact, the minimum change in a cross-sectional area of the tensile specimen owing to the gas dissolution is similar to the sorption rate (Figure 14). Specifically, the reduction in the network density affects the tensile strength and strain hardening modulus [12]. Because the region where the gas is absorbed is an amorphous region of the polymer, the change in the volume of the specimen is directly related to the network density of the polymer chain. Although a deviation occurs, the sorption rate and the area expansion rate of the gas show a similar variation pattern within the range of 0.9%–1.3%: as the amount of gas dissolved increases, the volume increases. When left at atmospheric pressure, the gas escapes through diffusion, and the volume decreases again. This increase in volume results in a decrease in the network density of the polymeric material, which can explain the decrease in strength shown in Figure 11, Figure 12 and Figure 13.

## 4. Conclusions

This study focused on the mechanical properties of a solid-state polymer–gas mixture, an intermediate product of the batch foaming process. A mixture of APET and CO_2_ was formed through diffusion based on the difference in partial pressure using a high-pressure vessel. The behavior of the characteristics was confirmed by measuring the impact and tensile strengths of the polymer–gas mixture. For the impact strength, high-pressure CO_2_ was adsorbed into the APET specimen through diffusion owing to the difference in partial pressure, forming a polymer–gas mixture, which caused an increase in the volume and impact strength. The tensile strength changed as a result of the dissolved CO_2_, and the extent of changes in the mechanical properties decreased as the amount of gas dissolved decreased. Detailed conclusions regarding the mechanical properties of the impact and tensile strengths are as follows:

### 4.1. Impact Strength Change through CO_2_ Adsorption

1. Based on an observation of the sorption rate and volume expansion, the free volume can be confirmed through the part with sorption rate that does not cause a change in volume, and the volume expansion is linearly related to the sorption rate in the part having a sorption rate of 2% or higher.
2. It was also confirmed that the CO_2_ improves the impact strength of the APET by volume increase, by acting as a cushion. Also, the plasticizing effect of CO_2_ works on the sample, even in a solid-state polymer sample. The rapid increase in the impact strength is equal to a sorption rate of 2%, which is the starting point for the volumetric expansion. A maximum value 956% higher than that of the reference specimen at a sorption rate of 2.85% was observed.
3. It was confirmed that CO_2_ dissolved in the polymer has the effect of increasing the impact strength in a specific sorption rate.

### 4.2. Tensile Strength Change through CO_2_ Adsorption

1. As the amount of dissolved CO_2_ increases, the tensile strength decreases. In this experiment, the sorption rate was changed by varying the exposure time (sorption time) to high-pressure CO_2_, and the tensile strength was decreased with this change.
2. The amount of CO_2_ dissolution affects not only the tensile strength but also the deformed stress-strain curve formation of APET. In particular, when CO_2_ was dissolved over a certain degree of sorption rate (6.67%), a homogenous deformation occurred without the occurrence of necking on the stress–strain curve.
3. The amount of dissolved CO_2_ and the tensile strength have a linear correlation (decrease in tensile strength ∝ sorption rate), and the extent of reduction in the tensile strength increases as the amount of dissolved CO_2_ increases.
4. Changes in the mechanical properties from CO_2_ dissolution are related to the network density of the amorphous region of the polymeric materials. As the amount of dissolved gas increases, the increase in area and volume increases. In addition, the sorption rate and volume of the gas decrease again as the retention time (desorption time) increases after sample removal from the high-pressure condition, and thus the tensile strength is also close to the value of the reference specimen.

## Figures and Tables

**Table 1:** Experimental conditions for studying changes in impact strength.

Material	APET	
Adsorption Condition	Gas Pressure (MPa)	5
	Vessel Temperature (°C)	23
	Adsorption Time (h)	1–120
Desorption Condition	Desorption Pressure (MPa)	0.1
	Desorption Temperature (°C)	23
	Desorption Time (min)	4

**Table 2:** Experimental conditions used for studying changes in tensile strength.

Material	APET	
Adsorption Condition	Saturation Pressure (MPa)	6
	Saturation Temperature (°C)	23
	Saturation Time (h)	4/8/12/16
Desorption Condition	Desorption Pressure (MPa)	0.1
	Desorption Temperature (°C)	23
	Desorption Time (min)	4
